# Community Management of Acute Malnutrition (CMAM) in Odisha, India: A Multi-Stakeholder Perspective

**DOI:** 10.3389/fpubh.2018.00158

**Published:** 2018-06-19

**Authors:** Sanghamitra Pati, Sandeep Mahapatra, Rajeshwari Sinha, Sandipana Pati, Satya N. Samal

**Affiliations:** ^1^Department of Health Research, ICMR Regional Medical Research Centre, Bhubaneswar, India; ^2^Indian Institute of Public Health, Public Health Foundation of India, Bhubaneswar, India; ^3^Independent Researcher, New Delhi, India; ^4^Department of Health and Family Welfare, Government of Odisha, Bhubaneswar, India; ^5^Department of Health and Family Welfare, Directorate of Health Services, Government of Odisha, Bhubaneswar, India

**Keywords:** community management, severe acute malnutrition, CMAM, nutrition interventions, malnutrition, public health, primary care

## Abstract

India remains home to nearly one-third of the world's children with severe and acute malnutrition (SAM). The present study looks at the function and implementation of a Community Management of Severe Acute Malnutrition (CMAM) programme for treatment of children with SAM in Odisha, an Indian state. A cross-sectional study design using qualitative techniques with direct observation of process and infrastructure was adopted to explore the views of stakeholders on the programme implementation. The study focuses on Kandhamal, a district in Odisha, and was conducted during June–August, 2015. Of the district and community level stakeholders involved in CMAM programme, 49 were selected as study participants using purposive sampling. In-depth interviews were conducted to obtain relevant information. Data was analyzed using data analysis software, atlas.ti version 7. The analysis demonstrated the overall acceptability, feasibility and economic viability of the programme. Additionally, the study identified several enablers (such as good response from child, village leadership involvement, multisectoral participation etc.) and barriers (such as limited awareness, increased work load, irregular staff payment etc.) linked to programme implementation. Interactions with beneficiaries and stakeholders also provided the real picture on the ground. The study emphasizes the need for stakeholders to work responsibly and in unison, and need for beneficiaries to accept, participate and contribute to the programme. In view of maximum impact, the study recommends that CMAM programmes be implemented with existing primary healthcare facilities. The study also outlines future scope for policy-level interventions and support to ensure sustainability of this healthcare delivery model.

## Introduction

Nutrition for children has been a global priority for many years now. However, most countries regularly track only limited nutrition indicators while even fewer pay attention to the range of manifestations of malnutrition. As a result, nutrition policies and strategies are either only instruments, inadequately tailored to address multiple forms of malnutrition or too broadly devised, to offer concrete guidance on programme levers that could be organized to address nutrition problems.

In the last decade, India has made impressive strides in improving a number of health indicators. At the same time, the decade has also witnessed increased inequality and uneven progress, especially on the fronts of health, education, and child nutrition. For example, wasting (low-weight-for-height), along with its increased risks for morbidity has remained a major health problem. Globally, wasting accounts for 4.7% deaths in children below 5 years of age with lower and middle income countries such as Nigeria, Pakistan and India reporting a wasting prevalence of >10% throughout the year (1). The number of children <5 years of age with wasting in India has gone up from 19.8 to 21% as per the fourth National Family Health Survey (NFHS)2015–16 (2). Wasting in children <5 years is the most common indicator of Severe Acute Malnutrition (SAM) and constitutes almost one-third of the global burden (3). While Sustainable Development Goal 2.2 aims to end all forms of malnutrition by 2030 and achieve internationally agreed targets on stunting and wasting in children, data on increasing wasting trends in India is a cause for worry despite several number of child health programmes in the country.

Odisha is one of the coastal states in the eastern region of India. The state so far has been on the weaker side of desired nutrition outcomes. For example, the children <5 years with wasting in Odisha has increased from 19.6% in NFHS 3–20.4% in NFHS 4 (2). The state government has been implementing various initiatives to address SAM across the state. Although the principle strategy remains inpatient care through Nutritional Rehabilitation and Malnutrition Treatment Centers (4, 5), there is a growing consensus that community-based management of acute malnutrition (CMAM) is crucial approach for achieving widespread, effective coverage and treatment of all children with SAM (6, 7). CMAM programme encompasses four key components, which include community outreach activities, community based management of children with SAM without complications, inpatient care of children with SAM having complications and community based management of children with moderate acute malnutrition (8). The approach seeks to shift the treatment of a SAM-afflicted child from medical facilities to within the community, at the same time providing economic and logistic advantages. Treatment of SAM is also able to achieve a much wider coverage and access, timeliness and appropriate care as compared to inpatient treatment approaches. Key elements that facilitate CMAM are use of mid-upper arm circumference (MUAC) method for simplified, accurate, inexpensive and timely screening; use of ready-to-use therapeutic foods (RUTF) or nutrient dense foods, which can be prepared and used safely at home enabling home-based treatment; and use of a classification system that divides SAM afflicted patient into those with and without medical complications (9). Evidences from existing CMAM programmes have shown that such community-based approaches have been successful with improved recovery and mortality rates.

In 2015, the Odisha government piloted CMAM in tribal-dominated Kandhamal district to reduce malnutrition-related complications among children. It assumed significance as the district recorded the highest levels of under 5-year child mortality in the state in 2012–13. The programme was launched through the existing system of Anganwadi Centres, rural mother, and child care centers, under the Women and Child Development and Health and Family Welfare departments. A standard CMAM programme consists of treatment sites closer to the community where children with uncomplicated SAM can be seen weekly, and an inpatient facility that admits only children with SAM plus associated medical complications that require specialist medical attention and keeps them only until they recover enough to continue treatment as outpatients in the community.

However, a major apprehension to the widespread adoption of this strategy in India including Odisha, is that the vast majority of evidence on the effectiveness of CMAM comes from non-Indian settings, where CMAM programmes have resulted in reducing malnutrition among children. This has not been explored very extensively in India and very few reports on the success of CMAM in India are documented (10). This is the first time, a paper highlights evidence on the implementation process of the CMAM programme gathered from Odisha and goes beyond understanding the technical details and the programme's effectiveness to discuss the perspectives of the mothers and care givers on the programme. The specific objectives of the study are to examine the perspectives of various stakeholders involved in implementation of the CMAM programme, to assess enablers and barriers faced by them with regard to programme implementation, and also identify suggestions for improving the CMAM implementation process. The study gives a unique opportunity to draw a comparison between the provider and recipient perspectives. It is important that stakeholders work in unison and have a clear understanding of their functions in such programmes while beneficiaries willingly accept the programme. Further, the paper also makes specific recommendations drawn from the in-depth interviews of various stakeholders to strengthen CMAM.

## Methods

### Study design

A cross-sectional study design using qualitative techniques with direct observation of process and infrastructure was employed to explore the views of various stakeholders in the CMAM programme.

### Study setting

The study was carried out in Kandhamal district of Odisha. The district has 2 subdivisions, 12 blocks, 153 Gram Panchayat (village councils under blocks), 2,546 villages and 172,022 households. As per the 2011 census, Kandhamal had population of 733,110, of which male and female were 359,945 and 373,165 respectively.

### Study duration

The present study was conducted during June-August, 2015.

### Study participants and framework

The study participants involved in the CMAM programme included stakeholders from state, district, block and community level. As this was a process implementation study of a pilot project initiated by the government, the participants were selected using purposive sampling using the principle of maximum diversity. They were interviewed at the intervention site as the sampling unit.

It was ensured that participants represented multiple levels of stakeholders involved in CMAM implementation. Therefore, all stakeholders involved in the programme, for example, children, mothers, health department officials in charge of programme execution and management as well as ground level healthcare providers were considered. The final inclusion of the participants was based on individual interviews followed by their willingness to participate in the study. A total of 49 study participants were included.

The sample included Chief District Medical Officer, Medical Officer, District Social Welfare Officer, District Programme Manager, District Nutrition Manager, Medical Officer In-Charge, Block Programme Manager, Public Health Emergency Officer, Health Worker Female, Accredited Social Health Activists, Anganwadi Workers, Child Development Project Officer, Lady Supervisor, Non-government organization Staffs, Panchayati Raj Institution members and parents of children <5 years who were beneficiaries of the CMAM programme.

### Data collection tools

In-depth face-to-face interviews were conducted using a discussion guide, which covered the following range of topics: socio-demographic information (age, general education, administrative positions, and working experiences), details of services provided in the CMAM programme, experiences of beneficiaries, perception about effect of the programme on children, involvement and working of various health workers in the programme and suggestions to strengthen the programme. Respondents were asked for their views on major factors which may be responsible for the success of the CMAM scheme. The discussion guide was piloted before using it in the study. After piloting, necessary corrections were made and a final semi-structured questionnaire was prepared (Supplementary Material). The interviews were recorded and then transcribed verbatim and translated to English. Direct observation of the processes and infrastructure for the effective implementation of the programme was also done during interview.

### Data analysis

We used qualitative data analysis software, atlas.ti version 7, to code, analyze thematically and draw inferences from the interviews (11). The process of generating themes was deductive. We further identified coded, recoded and classified themes by examining regularities, convergences and divergences in the data. In order to ensure the quality of results, two researchers (authors of the paper, Sanghamitra Pati and Sandeep Mahapatra), conferred on the analysis separately by coding text and generating themes and sub-themes and then comparing output. In addition, the results were circulated to respondents to check that the findings had fidelity with their perceptions and experiences, and minor adjustments were made.

### Ethical consideration and consent

The Ethics Committees of the Indian Institute of Public Health, Bhubaneswar approved the protocol for the study. All respondents were informed of the study objective and of their freedom to participate or withdraw from the study at any point. Respondents gave written consent to be interviewed and for the interviews to be audio-recorded. Recordings and transcripts were coded so that the origin of the respondents could not be identified. All steps were taken to ensure that confidentiality and anonymity are maintained at all times.

## Results

### Programme details

#### Detection during village health nutrition day sessions

The present study was aimed at assessing the implementation of a pilot scale CMAM programme in Odisha. An assessment of “when” the cases were detected under this programme revealed that all cases were detected at the village level during the Village Health Nutrition Day session. They were enrolled in the programme after rechecking by concerned staffs as per criteria fixed in the programme. The cases were enrolled immediately within two to three days. The whole process was done consultatively by both CMAM staff and the local medical officer. Most of the programme staff mentioned that they were satisfied with the cooperation extended by the parents. According to the programme personnel, initially, there was less response from the parents; which gradually increased later. The staff attributed it to increasing awareness among the beneficiaries about this programme.

“*My child was detected during Village Health Nutrition Day before enrollment in the programme” - Mother of child with SAM*

#### Treatment modalities

**Figure 1:**
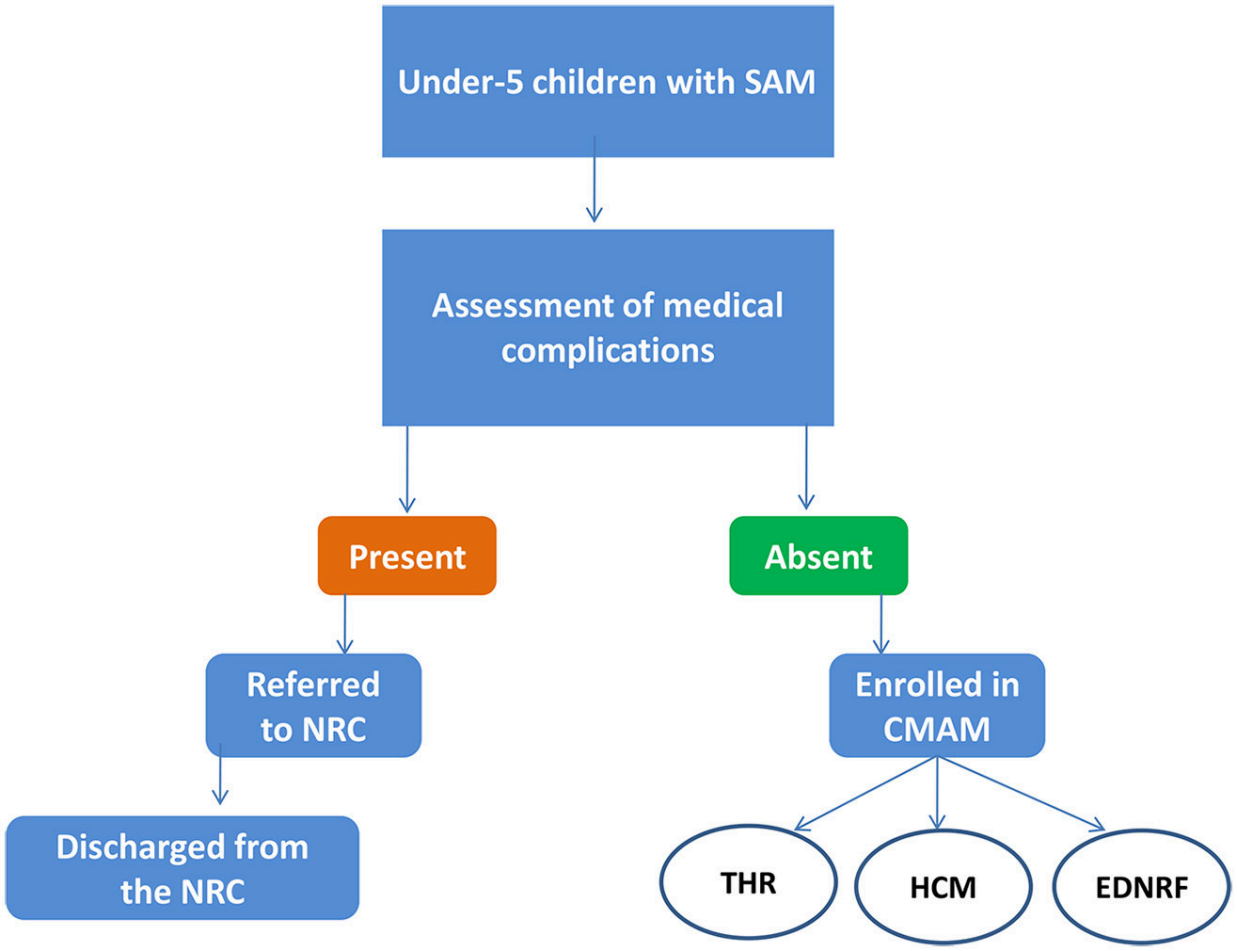
Management of Severe and Acute Malnutrition (SAM) in Community-based Management of Acute Malnutrition (CMAM) programme.

The cases were mainly treated with the antibiotic amoxicillin, deworming agent albendazole, Vitamin–A and iron folic acid syrup along with nutritional rehabilitation. Nutritional treatment followed a 3-arm modality with (i) Modified Energy Dense Nutritional Food (EDNRF) (ii) Modified Hot Cook Meal (HCM) and (iii) Modified Take Home Ration (THR) (Figure 1). In EDNRF, semi-solid food is provided for consumption through a dose of 1–2 pouches per day, as per the age and weight of child with SAM. In the THR, pouches of solid dry ghee mixed *chhatua* were given, with instructions that the pouches were to be prepared with warm water and consumed. The HCM diet included freshly prepared food at Anganwadi Centres (AWC) as per the menu, for which the child under treatment was also required to visit the AWC unlike the THR and EDNRF which could be administered at home by the mother herself. Initially the HCM, consisting of *sujikhiri, chudakhiri, magajaladoo, rice* with *mixed vegetable curry* and *dal*, was started in Tikabali block and the child had to consume it six times a day; but due to operational problems, it was later shifted to Daringibadi block and the frequency was reduced to four times a day. However, in Daringibadi block, hygienic conditions during preparation could not be maintained due to the non-availability of Anganwadi Workers and AWCs. Unsurprisingly, the mothers denied taking their children to AWCs for diet four times a day due to the widespread and hilly terrain of the area.

#### Child response to the treatment

All stakeholder interviewed were of the opinion that both the MUAC and body weight of the child with SAM improved during the course of the programme. Many of the children were faced with diarrhea after initial treatment due to indigestion which subsided by time. The weight gain by the child in the first week was less but increased in the latter part of the programme. The initial slow weight gain may possibly be attributed to diarrhea, less appetite in the child or consumption of less diet.

#### Role of NGO staffs, anganwadi workers, and accredited social health activists

As per the opinion of block and district level staff, the role of non-government organization (NGO) staff and Anganwadi Workers (AWW) were impressive. The NGO staff cross-checked the MUAC and weight of the child. They also took care of the supply part of logistics at the AWC. All of the AWW visited the child's house regularly. They also counseled mothers with the help of NGO staffs regarding the Infant and Young Child Feeding, breast feeding, hand washing, personal care, and hygiene practices. Most of the AWW agreed that the programme increased their workload but since they lived in the village, they could handle the same. The Accredited Social Health Activists (ASHAs) did the blood testing and supplied medicine under direct supervision of female health worker.

“*When elder people demand for the diet/nutrient supplied by the government, we are giving it to them.” - Panchayati Raj Institution Members*

“*We are unable to supervise always whether the baby taking the Supplementary Nutrition Programme or not” - Anganwadi Workers*

#### Monitoring and reporting in the programme

The quality of the programme was mainly reviewed in the sector meeting, project meeting and joint convergent meetings of Health and Integrated Child Development Services (ICDS). The district and block level programme officers supervised through home visits, direct observation, crosschecking reports for MUAC measurement of SAM children, and talking to their mothers during Village Health Nutrition Day. The reports were prepared by the AWW and submitted to the Child Development Project Officer's office. The NGO was mainly involved in the report validation and analysis. Some AWWs reported that initially they did not have the competency for reporting and had taken the help of peer AWW and NGO staff. Their skills improved through the support of peer workers and NGO.

The AWWs reported that there were no annual action plans prepared but they followed the weekly action plans as per the screening report in that week. The reports were kept at three places: beneficiary, AWW and block office. The block and district level staff informed that they receive feedback from the grass root level workers and the action is taken accordingly. The grass roots level workers also opined that the supervision by higher authorities helped in building their confidence.

“*The NGO staff helped us in the initial stage of project in report writing” – an Anganwadi Worker*

#### Follow up after discharge from the programme

The SAM children were discharged from the programme after 8 weeks, if they reached a MUAC of 115 mm. If the concerned child did not attain this desired outcome, they were again enrolled for another 8 weeks. They were further followed by regular visits by the AWW and ASHA, during which they were counseled along with weekly MUAC and weight checkups. During discharge, all mothers were advised to report to AWW or ASHA in case of problems.

#### Supply of nutrients and other logistics

It was reported that the supply of the nutrients was not sufficient at the initial stages but became adequate later. It was also confirmed that blood testing kits and other medicines were available with all ASHAs and reporting formats were available with most of the AWWs.

### Constraints faced by stakeholders during implementation of the programme

The main problems identified in the programme implementation were the vacancy in the AWW workforce and non-availability of their own AWC buildings. One of the district level staff and many of the ICDS supervisors opined that the AWWs had limited efficiency in providing the quantity of HCM as per the child's weight. In hard to reach areas, the mothers did not bring their children for the diet four times a day. Due to the non-availability of AWCs, the hygiene condition during the preparation of the diet was also compromised. The THR was often not consumed by the child if the mother was working. In the harvesting season, the cooperation by family members was not good. The AWWs expressed their work overload and had to remain in the center till night as well as work on Sundays. As per the CMAM guideline of Bangladesh, a child will not be enrolled into CMAM if his/her MUAC is not <115 mm, even though it comes within the red zone of the WHO growth chart (8). Non-enrollment of children therefore prevented access to services for many children. All the field staff, the ASHAs, AWWs and helpers reported not receiving timely remuneration and some instance of non-payments were also recorded.

“*There is no availability of Anganwadi Workers and Anganwadi Centres in many places of Daringibadi block, so preparation of Hot Cook Meal was a problem” – District Nutrition Manager*

“*Non-enrollment of child if MUAC more than 115 mm, even he comes in red zone of WHO growth chart, prevents many child from getting the benefits”- District ICDS Officer*

### Enabling factors for the success of the programme

All stakeholders interviewed mentioned that the children do achieve the desired weight after treatment and the MUAC also increases. The AWWs were of the view that the specially planned diet drastically improves the nutritional status. The nutritional supplements were available free of cost. The HCMs prepared at the AWC were mixed with vegetables to enable children to obtain balanced nutrition at the same time. Most of the stakeholders agreed that the child with SAM was identified at the right time before complications started, and the treatment was given in a domiciliary setup at the family level. The programme officer also reported a decrease in load of the Nutrition Rehabilitation Centers due to initiation of CMAM and the family members received appropriate care. All interviewees agreed that the involvement of Panchayati Raj Institution members, self-help group members, and village level leaders also gave positive impact to the programme.

“*My child was so weak that he was unable to walk; but after treatment he can walk properly” - Mother of child with SAM*

“*The treatment at home gives a social and moral support to the family members” - Chief District Medical Officer*

“*The supply of cost-free diet is beneficial to child which a poor family cannot afford” - Mother of beneficiary*

The overall framework for barriers and enablers identified from the situational analysis of the government initiative has been summarized in Table 1.

**Table 1:** Barriers and enablers framework.

	**Barriers**	**Enablers**
Community level	Limited awareness Take Home Ration not prepared by other family member	Response of child is good Domiciliary treatment gives moral and social support
Government level	Vacancy of AWWs Non-availability of AWC building Strict inclusion criteria of MUAC Work overload of AWWs Non-payment/delay in disbursing incentives	Identification at right time Involvement of Panchayati Raj Institutions/Self-help Groups/village leaders Multi-sectoral involvement
Recommendations	Discharging criteria should be increased to MUAC 125 mm Regular and increased refresher trainings to grass root level workers Payment processes should be regularized and incentivized to increase interest of the workers Better logistics for the programme should be procured (for e.g., digital weighing machine) Village Welfare Committee or Gaon Kalyan Samiti should be involved in Information Education and Communication or Behavior Change Communication activities for community mobilization and regular discussion regarding CMAM during Village Health Nutrition Day	

## Discussion

Childhood malnutrition is one of the major public challenges in India. Management of SAM requires adept public health measures to enhance dietary quality and quantity. The objective of this study was to assess the current situation of pilot CMAM implementation in Kandhamal district in Odisha.

Our assessment of the CMAM programme suggests that programmatic implementation was as per the desired objectives. CMAM was an implementable and acceptable strategy, as reported by the beneficiaries and stakeholders involved. The assessment of barriers and enablers provides useful information on the health system preparedness of the district implementing CMAM. At the same time, it is equally important to have an understanding of the existing strengths as well as the areas that need further strengthening. During the course of the study, suggestions were also received by the respondents. The suggestion not only provides an assessment of on-the-ground challenges and limitations but also aids the identification of factors that need to be improved for creation of an enabling environment for programme implementation. A study on Village Health Nutrition Day implementation by the authors has also previously identified similar health system challenges (12).

Most of the evidence on CMAM effectiveness is generated from African settings, such as Malawi, Ethiopia, Sudan, Nigeria, Ghana, Mozambique, Kenya, Zambia etc. (13, 14). A few Asian countries like Bangladesh and Sri Lanka have also explored the CMAM effectiveness (15, 16). In the Indian context, there is a need to generate more evidence and case studies. The first commercial CMAM programme in India was carried out in Bihar in 2009 for children with SAM and achieved lower mortality and high cure rates (8). Assessment studies in the Indian states of Chandigarh, Jharkhand and Uttar Pradesh suggest the need for incorporation of community based programmes as a key component of the continuum of care in children with SAM (5, 17, 18). The present study adds to the gradually growing evidence base on the effectiveness of community-based programmes in India.

The CMAM model has several advantages over inpatient treatment and facility based treatment settings. For example, it provides a framework for integrating public health responses and various other interventions designed to reduce the incidence of malnutrition. The decentralized nature of such a programme enhances community participation and helps increase access to treatments. CMAM programmes for treatment of SAM have also been reported to minimize costs to families and to be highly cost effective (19, 20). In view of the community-based outreach and maximum impact, it is time that CMAM programmes be implemented in integration with primary healthcare whereby children are able to receive care through the already existing mechanisms within the primary healthcare system. In the present study, parents accessing SAM treatment through CMAM reported incurring considerably lower expenses. However, despite many programmatic benefits, the absence of national level guidelines regarding CMAM makes scale up of SAM treatment difficult. Implementation of an effective public health approach for addressing SAM in India will require significant attention by policy makers to develop situation-specific, customized and sustainable models of care.

## Limitations

Data obtained in this study could only enumerate the barriers, enablers, and suggestion to improve. Since purposive sampling was used, this might not represent the whole population.

## Conclusion

As home to nearly one-third of the world's children with SAM, India's approach to treating SAM has relied almost exclusively on inpatient care, while community-based approaches have not been explored much or implemented fully. This paper describes the function of a CMAM programme in one of the tribal-dominant districts of Odisha, where CMAM has been an effective and economical strategy. CMAM therefore appears suitable for integration into mainstream healthcare system delivered at community level. However there is still some room for improvement.

## Author contributions

All the authors have made a substantial contribution to the conception and design and/or the analysis and interpretation of data, drafting the article as well as revising it critically for intellectual content.

### Conflict of interest statement

The authors declare that the research was conducted in the absence of any commercial or financial relationships that could be construed as a potential conflict of interest.
