# Supplementary material for: Community Management of Acute Malnutrition (CMAM) in Odisha, India: A Multi-Stakeholder Perspective
Source: Front Public Health. 2018 Jun 19;6:158. doi: 10.3389/fpubh.2018.00158 (PMC6018096; doi:10.3389/fpubh.2018.00158)
Supplement: Supplementary file 1 [file Data_Sheet_1.DOCX]

**Appendix: Semi-structured questionnaire for stakeholders**

**1. Sub-Centre level /AWW/ASHA/Community level Questionnaire for Community Management of Acute Malnutrition (CMAM) in Odisha, India: a Multi-Stakeholder Perspective**

Date of Interview ------------------------------------- Respondent ID--------------------------------Name:…………………………………………….Designation………….

Name of the Block-----------Name of the Sub-centre: ---------------District: Kandhamal.

1. How many children under SAM are there in your area?
2. When did you diagnose these cases (all cases)?
3. When did you enroll these cases in CMAM (all cases)?
4. How you know that it is a case of SAM?
5. Why do you consider it to be under CMAM?
6. How should a SAM child be treated under CMAM in your area?
7. How are the NGOs (Valid International) helping you in this programme?
8. Did the parents of children co-operate with you in the programme?
9. How is the child responding to your programme?
10. What type of support is provided to the child’s family after the child is discharged from the programme?
11. How do you enter the information in the reporting form?
12. Do you use specific reporting forms for sending information?
13. Have you submitted complete reports timely in last two weeks?
14. If *No,* why was the report delayed or incomplete for submission?
15. How does this service affect their overall workload?
16. Have you ever been trained on CMAM?
17. If *Yes*, when was the last training attended?
18. How often have you been supervised by your higher authority?
19. Do you get any feedback from the higher authority? If Yes, how often:
20. Most of the time
21. Sometimes
22. Never
23. What are the constraints in working for CMAM (If any)?
24. What are your suggestions to overcome these constraints?
25. What are the good things you found from this programme and why do you feel CMAM is important?
26. Any suggestion to improve CMAM.
27. How many among these children were under the Supplementary Nutrition Program?
28. Was the child taking the Supplementary Nutrition?
29. If *No* then what steps according to you should be taken to ensure that Supplementary Nutrition is consumed by children?
30. How do you know that the children were taking Supplementary Nutrition?
31. Direct Observation: - Match the information from register and reporting forms for last 2 weeks :

Are the logistics adequate and supplied regularly?

|  | Adequate (Yes/No) | Regular (Yes/No) |
| --- | --- | --- |
| Forms |  |  |
| Registers |  |  |
| Rapid Diagnostic Kit |  |  |
| Nutrients |  |  |

**2. Beneficiary level Questionnaire for Community Management of Acute Malnutrition (CMAM) in Odisha, India: a Multi-Stakeholder Perspective**

Date of Interview------------------------------Respondent ID---------------------------------- Name:……………………………………..Village ……………………………..

Name of the Block-------------------------------District: Kandhamal.

1. How many of your children are under CMAM?
2. When was your child diagnosed as SAM?
3. When was your child entered in to CMAM?
4. How is your child treated under CMAM?
5. Are the staffs like Anganwadi Workers co-operative?
6. How does your child respond to the programme?
7. What are the problems that you faced in CMAM (If any)?
8. What are your suggestions to overcome these constraints?
9. What are the good things that you found from this programme and why do you feel CMAM is important?
10. Any suggestions to improve CMAM.
11. Do you belong to Above Poverty Line (APL)/Below Poverty Line (BPL) family?
12. Are you getting nutrition under Supplementary Nutrition Program?
13. If *Yes*, are you getting this nutrition regularly or not?
14. Was the child taking the Supplementary Nutrition?
15. If *No*, then why and what steps, according to you, should be taken to ensure the Supplementary Nutrition is consumed by your children?
16. What is the quality of nutrition received under Supplementary Nutrition Program?
17. How many children do you have?
18. What is the order of this child?

**3. BLOCK level / District level / State level Questionnaire for Community Management of Acute Malnutrition (CMAM) in Odisha, India: a Multi-Stakeholder Perspective**

Date of Interview------------------------------Respondent ID---------------------------------- Name:……………………………………..Designation……………………………..

Name of the Block-------------------------------District: Kandhamal.

1. How many CMAM units do you have under your jurisdiction?
2. Do all your reporting units submit their report regularly as regards to completeness and timeliness? If No, what initiative have you taken for regularization?
3. Do you have any standard case definitions for cases to be taken regularly in the CMAM?
4. If No, what procedure do you follow for defining cases under programme?
5. How do you assess the quality of your unit in CMAM?
6. Do you have any standard case management protocol under CMAM? If No, what you do?
7. Do you have blood testing facility for all cases? If No, what is the constraint?
8. Do you have annual action plan for the programme? If No, what are the constraints?
9. How do you review and monitor the CMAM work?
10. Are the nutrients and logistics supplied in adequate quantity and regularly for program for all cases
11. Adequate & regular
12. Not adequate but regular
13. Adequate but irregular
14. Not adequate & not regular
15. How do you analyze and interpret the data using CMAM reports?
16. No. of staffs trained on CMAM?
17. Do you get any feedback from the field staffs?
18. What action are you taking as per their feedback? How are you taking as per their feedback?
19. Do you get any feedback from the higher authority?
20. Most of the time

b. Sometimes

c. Never

1. What are the constraints in working for CMAM (If any)?
2. What are your suggestions to overcome these constraints?
3. What is the important and good thing you found in CMAM?
4. Why do you feel CMAM is important?
5. Any suggestion to improve CMAM?
